# Geriatric rehabilitation center outcomes after successful weaning from extremely prolonged mechanical ventilation in older adults

**DOI:** 10.3325/cmj.2024.65.431

**Published:** 2024-10

**Authors:** Igor Kisil, Yuri Gimelfarb, Arie Soroksky

**Affiliations:** 1 Medical-Care Hospital, Bat Yam, Israel; 2 AMHC, Bat Yam, affiliated with the Faculty of Medicine, Tel Aviv University, Israel; 3 WMC, Holon, affiliated with the Faculty of Medicine, Tel Aviv University, Israel; * The first two authors contributed equally.

## Abstract

**Aim:**

To determine the rate of and predictive factors for postacute in-hospital survival of older adults after successful weaning from extremely prolonged mechanical ventilation (e-PMV).

**Methods:**

This pilot retrospective study involved patients who were weaned from e-PMV in Bayit Balev Geriatric and Rehabilitation Center, Maccabi Health Services, Bat Yam, Israel between January 2010 and May 2022. In-hospital survival was measured.

**Results:**

Out of 488 patients (of all ages) treated in our geriatric rehabilitation center (GRC) during the study period, only 181 patients aged 65 years and older were conscious and were candidates for weaning from e-PMV. Seventy-three patients (40.3%) were weaned and therefore recruited to this study. Six patients (8.2%) were weaned but died before discharge. Out of the 67 (91.8%) who were alive and weaned, 18 (24.7%) were decanulated and discharged to their homes, and the remaining 49 (75.3%) were weaned and discharged from our GRC with tracheostomy canula to other long-term care institutions. Univariate analysis showed that in-hospital survival was significantly dependent on age and the presence of tracheomalacia, both in a time-independent and time-dependent manner. In a time-dependent (adjusted) multivariate analysis, there were no independent predictive factors for in-hospital mortality.

**Conclusions:**

A dedicated GRC team can wean a substantial number of patients after e-PMV and return them to their community and even homes.

Prolonged mechanical ventilation (PMV) is defined as ventilator dependence for ≥21 consecutive days, for ≥6 hours per day (1,2). Approximately a tenth of adults admitted to intensive care units (ICU) require PMV (3). These patients demand a huge amount of acute care resources, requiring a coordinated multidisciplinary approach to optimize outcomes (4). PMV is associated with high morbidity, mortality, and poor functional status (4). To minimize mortality and morbidity, and contain health care costs, it is essential to identify patients at high risk for PMV and to implement early interventions (4-6). Otherwise, failed weaning attempts are associated with high morbidity, mortality, and other adverse outcomes (6).

In Israel, during the last decades, numerous hospitals for long-term care have been established for chronically ventilated adults. The cost of care in these facilities is lower than that for care in the ICU. Most individuals in geriatric and rehabilitation centers are hemodynamically stable older adults who require, among others, PMV (7) or even extremely PMV (e-PMV) after acute illness (8).

Table 1 summarizes demographic information and outcomes of adult (both weaned and non-weaned) patients treated post-ICU by PMV across all studies available in the literature. Postacute in-hospital survival in adult population after PMV ranged between 36.5% (9) and 91.0% (10,11).

**Table 1:** Summary of studies assessing post-intensive care unit mortality rates of adult patients treated with prolonged mechanical ventilation (PMV≥21 days), both weaned and non-weaned*^†^

Reference	Facility and MV duration. D	Number of participants	Age, years	In-hospital mortality rates, n (%)	Predictive factors	Discharged, n (%)	Discharged directly home, n (%)
Aboussouan et al, 2008 (19)	PCU, >21	N = 113 weaned and not weaned	Median = 65.0 IQR 47-75 Range 15-85 Mean = 60.0 SD = 17.0	33 (29.2)	NA	80, of them 52 (65) weaned	10 (12.5)
Wu et al, 2009 (13)	RCC, ≥21	N = 736 weaned	Mean = 72.5 SD = 15.4	111 (15.1)	NA	625 (84.9)	NA
Hui et al, 2010 (11)	RCW, Median = 166.0 Range 34-1329 IQR 100-291	N = 243 weaned and not weaned	In weaned: Median = 80.0 IQR 75-84 Mean = 78.3 SD = 9.9	NA	NA	In weaned: 67	23 (34.3)
Mamary et al, 2011 (16)	VRU, Mean = 55.0 SD = 42.7	N = 182 weaned and not weaned	Mean = 64.1 SD = 15.6	35 (19.0)	By logistic regression: -higher CCI; -higher APACHE II score; -lower albumin.	147 (81.0), of them 125 (85) weaned	66 (44.9)
Dermot Frengley et al, 2014 (9)	LTACH, ≥21	N = 540	≥65 Mean = 79.2 SD = 8.1	343 (63.5)	NA	197 (36.5)	—
		of them 121 weaned		36 (29.8)	NA	85 (70.2)	20 (23.0)
Davies et al, 2017 (10)	SWU, ≥21	N = 458, of them 330 weaned	Median = 61.0 IQR 50-71	41 (9.0)	NA	417 (91.0)	343 (82.3)
Sansone et al, 2017 (25)	LTACH, ≥21	N = 437 weaned and not weaned	Mean = 62.4 SD = 16.6	159 (36.0)	NA	278 (64.0)	68 (24.5)
Huang, 2022 (26)	RCC, ≥21	n = 357 men, of them 242 weaned; n = 217 women, of them 149 weaned	Mean = 71.46 Mean = 73.88	113 (31.7) 73 (33.6)	NA	189 (52.9) men 107 (49.3) women	NA
Current study	GRC, ≥21 Median = 104 Range 23-941 IQR 72.0-194.0 Mean = 145.5 SD = 132.2	N = 73 weaned	Median = 78.0 range 65-94 IQR 72.0-83.0 Mean = 78.0 SD = 7.6	6 (8.2)	By Cox regression: No	67 (91.8)	18 (26.9)

*Abbreviations: GRC – geriatric and rehabilitation center; HQS – high-quality studies; ICU – intensive care unit; IQR –interquartile range; LTVF – long-term ventilator facility; LTACH – long-term acute-care hospital; MV – mechanical ventilation; NA – not available; PCU – pulmonary care unit; PWC – prolonged-ventilation weaning center; RCC – respiratory care center; RCW – respiratory care ward; SWU – specialized weaning unit; VRU – ventilator rehabilitation unit; VWU – ventilator weaning unit.

†all the presented studies are retrospective.

However, no studies identifying the predictive factors for postacute in-hospital survival in a time-dependent manner have been found. Moreover, little is known about the rates of survival and potential predictive factors for survival among older adults after successful weaning from e-PMV in the postacute geriatric rehabilitation setting. We therefore examined the outcomes of 73 older adults requiring e-PMV who had been transferred from acute-care hospitals, particularly from ICUs, to our geriatric rehabilitation center (GRC) and were successfully weaned from e-PMV. The aim of our study was to determine the rate and predictive factors for postacute in-hospital survival of older adults after successful weaning from e-PMV.

## Patients and methods

### Study design

The medical records of ventilated patients admitted from acute care hospitals to our GRC between January 2010 and May 2022 were reviewed. The study was approved by the Helsinki committee of Bayit Balev Geriatric and Rehabilitation Center, Maccabi Health Services, Bat-Yam, and informed consent was waived.

### Setting

This study was performed in Bayit Balev Geriatric and Rehabilitation Center, Maccabi Health Services, Bat Yam, Israel. This is a 282-bed geriatric and rehabilitation community teaching center (GRC) with 36 beds for chronically ventilated patients. The facility provides respiratory therapy, physical therapy, occupational therapy, speech and swallowing therapy, and other clinical services. It is staffed by critical care physicians, internal medicine and rehabilitation specialists, and general practitioners. This facility is a typical and representative one in Israel, serves as a tertiary referral center for more than four million population in a metropolitan area of the central region of Israel (12), and has been described elsewhere (7,8).

### Participants

During the study period, 488 patients aged 22-94 years were hospitalized in our GRC. Out of them, 102 were weaned from MV. The criteria for successful weaning have been described elsewhere (8).

The participants were enrolled in the current study according to the following inclusion criteria: age 65 years and older, both sexes, successful weaning from PMV in the period of their admission into our GRC, with or without physical or mental comorbidity. Overall, 181 patients aged 65 years and older were ready to undergo a weaning process. Out of them, 73 were weaned and therefore recruited to the study.

### Pre-transfer procedure

We were contacted by physicians from referring hospitals who intended to transfer patients on PMV to our GRC. The attending physician in charge of the patient filled in a questionnaire inquiring about the history of illness, weaning attempts and strategy, and physiological data concerning the patient’s status. The patients were transferred to our GRC a few days after our initial contact with the referring hospital.

### Admission criteria

All admissions to our GRC represent transfers from acute care hospitals. Patients were accepted regardless of the severity of their illness provided they were stable for transfer. Patients receiving mechanical ventilation were required to have a tracheostomy. Patients were discharged after complex medical issues had been resolved and they had either been safely liberated from mechanical ventilation or multiple attempts at liberation from mechanical ventilation had been unsuccessful.

### Variables

Data on participants were collected prospectively. The etiology and duration of ventilator dependency before transfer to the GRC were obtained from outside hospital records and the transferring physician. Demographic and clinical data on admission to GRC were obtained from participants’ medical records.

The initial cause of respiratory failure leading to ventilator dependence was classified into four categories as follows: acute lung disease (eg, acute respiratory distress syndrome, resuscitation, pneumonia, pneumothorax, or pulmonary embolism), chronic lung disease (eg, chronic obstructive pulmonary disease, neuromuscular disease, or thoracic restriction), neurologic diseases, and miscellaneous (6,8,13).

Patients who failed spontaneous breathing trial were screened for tracheomalacia. Severe tracheomalacia was diagnosed if there was 70% to 100% occlusion of the distal trachea during passive exhalation. All bronchoscopic confirmations were done by a single otolaryngology specialist.

The endpoint event was defined as either in-hospital death in our GRC or discharge from our GRC with or without recurrent MV in our GRC after successful weaning from e-PMV. Participants’ ideal body weight (IBW) was calculated by using the Stewart equation, based on height and body mass index (14). In bed-ridden patients, the height was calculated from the patient’s knee height (15).

All participants received a protocol-directed weaning strategy, which has been described elsewhere (8). The outcome measure was in-hospital mortality derived from the database, according to which the patients were divided into survivors (ie, discharged from our GRC) and non-survivors (ie, died in our GRC). These two groups were compared in terms of demographic, anthropometric, and clinical variables.

### Statistical methods

Continuous variables are presented as medians and interquartile ranges (IQR), while categorical variables are expressed as frequencies and percentages. All characteristics were compared between the subgroups with a Mann-Whitney U-test or χ^2^ test, as appropriate. Each parameter was tested regarding its potential influence on in-hospital survival.

Because of the substantially prolonged process of participant selection with a median of 125 months, Cox’s proportional hazard regression model was used to determine independent predictors for in-hospital mortality. It was used for univariate and multivariate analysis (both time-independent and time-dependent). The multivariate time-dependent (adjusted) model was constructed including all variables with significance of less than 15% (*P* < 0.15) in univariate analysis. Regression analyses results were expressed as hazard ratios (HRs) with 95% confidence intervals (CIs). A two-tailed p value less than 0.05 was considered significant. Data analysis was performed with SPSS, version 20, software for Windows (IBM Corp, Armonk, NY, USA).

## Results

### Participants

In total, 73 elderly adults were recruited (mean age ± standard deviation [SD], 78.0 ± 7.6 years; range: 65 to 94 years). They were ventilated from 23 to 941 days (145.5 ± 132.2). Demographic, anthropometric, clinical data, and details concerning e-PMV, including the comparison between survivors (mean age 77.6 ± 7.4 years) and non-survivors (mean age 83.1 ± 8.4 years) are shown in Table 2.

**Table 2:** Characteristics of older adults admitted to our geriatric rehabilitation center (GRC) and successfully weaned from extremely prolonged mechanical ventilation (e-PMV) (N = 73)*

Characteristic	Died in GRC (n = 6)	Discharged from GRC (n = 67)	Total (N = 73)
Age, median years (IQR)	86.0 (77.0-89.2)	77.0 (72.0-83.0)	78.0 (72.0-83.0)
Male sex, n (%)	5 (83.3)	30 (44.8)	35 (47.9)
Cause of PMV, n (%)			
acute lung disease	3 (50.0)	36 (53.7)	39 (53.4)
chronic lung disease	1 (16.7)	10 (14.9)	11 (15.1)
neurologic disease	1 (16.7)	18 (26.9)	19 (26.1)
miscellaneous	1 (16.7)	3 (4.5)	4 (5.4)
Time from intubation to tracheostomy, median days (IQR)	17.5 (8.2-25.0)	22.0 (14.0-31.0)	21.0 (14.0-30.5)
Time from tracheostomy to admission in GRC, median days (IQR)	11.0 (6.0-21.2)	18.0 (12.0-29.0)	18.0 (11.5-27.5)
Days of MV prior to transfer to GRC, median (IQR)	27.5 (21.8-46.2)	44.0 (33.0-67.0)	43.0 (29.5-64.0)
Tracheal injuries^†^			
tracheomalacia, n (%)	2 (33.3)	0 (0.0)	2 (2.7)
other, n (%):	0 (0.0)	3 (4.5)	3 (4.1)
no, n (%)	4 (66.7)	64 (95.5)	68 (93.2)
Comorbidities			
diabetes, n (%)	1 (16.7)	27 (40.3)	28 (38.4)
dementia, n (%)	4 (66.7)	28 (41.8)	32 (43.8)
vegetative state, n (%)	5 (83.3)	39 (58.2)	44 (60.3)
CCI, units	8.5 (5.8-9.5)	6.0 (6.0-8.0)	7.0 (6.0-8.0)
Cooperation – partial or full, n (%)	3 (50.0)	34 (52.3)	37 (52.1)
Aspiration, n (%)	3 (50.0)	23 (35.4)	26 (36.6)
Height, median cm (IQR)	160.4 (146.5-177.2)	162.4 (152.1-170.7)	162.4 (152.0-170.7)
Knee height, median cm (IQR)	48.5 (45.0-52.8)	50.0 (45.2-53.7)	50.0 (45.0-53.0)
IBW, median kg (IQR)	64.4 (53.7-78.5)	66.0 (57.8-72.8)	65.96 (57.76-72.82)
MV method – VC-IMV, n (%)	6 (100.0)	50 (74.6)	56 (76.7)
Pressure support, median cmH_2_O (IQR)	15.0 (11.0-15.5)	12.0 (12.0-14.0)	12.0 (12.0-15.0)
PEEP, median cm H_2_O (IQR)	5.0 (5.0-5.0)	5.0 (5.0-5.0)	5.0 (5.0-5.0)
FiO_2_, median (IQR)	40.0 (40.0-45.0)	40.0 (40.0-40.0)	40.0 (40.0-40.0)
f, median breaths/min (IQR)	15.0 (12.0-22.0)	17.5 (15.0-19.0)	17.0 (15.0-19.0)
V_T_, median ml (IQR)	.45 (.38-.46)	.45 (.40-.50)	0.5 (.45-.5)
V_T_/IBW, median ml/kg (IQR)	10.2 (8.8-11.1)	10.0 (8.9-11.2)	10.0 (8.8-11.1)
RSBI, median breaths/min/L (IQR)	50.0 (39.5-52.6)	40.0 (32.3-47.4)	40.0 (32.7-47.5)
Weaning from O_2_, n (%)	0 (.0)	21 (31.3)	21 (28.8)
Weaning from catheter, n (%)	1 (16.7)	33 (46.2)	34 (46.6)
Weaning from zonda, n (%)	1 (16.7)	33 (46.2)	34 (46.6)
Decanulation, n (%)	0 (.0)	29 (43.3)	29 (39.7)
e-PMV duration – total, median days (IQR)	110.0 (75.2-162.5)	104.0 (72.0-197.0)	104.0 (72.0-194.0)
Length of stay in GRC, median days (IQR)	247.0 (179.0-417.8)	75.0 (51.0-158.0)	88.0 (51.0-186.0)^‡^
Duration of weaning from e-PMV, median days (IQR)	49.0 (27.0-64.5)	29.0 (18.0-49.0)	30.0 (18.5-51.5)
Duration from admission to GRC to complete weaning from e-PMV, median days (IQR)	82.5 (38.5-131.2)	54.0 (31.0-92.0)	55.0 (32.0-106.5)
Duration since complete weaning from e-PMV to endpoint event (death/ discharge/ rec MV), median days (IQR)	9.5 (7.0-52.8)	17.0 (10.0-37.0)	17.0 (9.0-36.5)
Recurrent MV – after weaning from ePMV, n (%)	6 (100.0)	2 (3.0)	8 (11.0)^†^
**Causes of death**			
sepsis, n (%)	2 (33.2)	–	
acute coronary event, n (%)	1 (16.7)	–	
multiorgan failure, n (%)	1 (16.7)	–	
tumor and metastases, n (%)	1 (16.7)	–	
ARF, n (%)	1 (16.7)	–	

*Abbreviations: ARF – acute renal failure; CCI – Charlson Comorbidity Index; f – breathing frequency; FiO_2_ – fraction of inspired oxygen; IBW – Ideal body weight; RSBI – rapid shallow breathing index; VC-IMV – volume control intermittent mandatory ventilation; V_T_ – tidal volume; PEEP – positive end-expiratory pressure; RSBI – rapid shallow breathing index.

†*P* < 0.001.

‡*P* < 0.01.

Compared with survivors, non-survivors had a higher prevalence of tracheomalacia (*P* < 0.001) and recurrent MV (*P* < 0.001), and a shorter length of stay (*P* < 0.010) in the GRC. Survivors and non-survivors did not significantly differ in terms of other variables, including tracheal injuries: 2 non-survivors had tracheomalacia, 1 survivor had granulation, and 2 had tracheal stenosis. Although the study period included the COVID-19 pandemic, no cases of SARS-CoV-2 were diagnostically detected among the study participants.

### In-hospital mortality

During the observation period, 6 (8.2%) of 73 participants died during the stay in our GRC (non-survivors), all of them with recurrent MV during the period of 4, 8, 9, 10, 19, and 191 days after weaning from e-PMV. Sixty seven (91.8%) of 73 participants were still alive and discharged (survivors) from our GRC (of them, 2 with recurrent MV during the period of 7 and 10 days after weaning from e-PMV).

### Independent predictors for in-hospital mortality

Univariate analysis showed in-hospital survival to be significantly dependent on age and tracheomalacia, in both a time-independent (Table 3) and time-dependent (Table 4) manner. Multivariate time-dependent (adjusted) regression analysis (Table 4) identified no independent predictive factors for in-hospital mortality.

**Table 3:** Univariate Cox’s proportional hazard regression analysis of potential predictors of in-hospital mortality in older adults (N = 73)*

Characteristic	HR	95% CI		*P* value
		lower bound	upper bound	
Age	1.13	1.008	1.26	0.035
Sex – male vs female	2.79	0.30	26.10	0.37
Cause of PMV				0.90
acute lung disease	1.23	0.24	6.42	0.81
chronic lung disease	1.65	0.18	14.86	0.66
neurologic disease	0.46	0.05	4.13	0.49
miscellaneous	1.20	0.14	10.54	0.87
Time from intubation to tracheostomy	0.98	0.92	1.04	0.45
Time from tracheostomy to admission in GRC	0.97	0.92	1.03	0.34
Days of MV prior to transfer to GRC	0.97	0.94	1.02	0.31
Tracheal injuries				0.09
tracheomalacia vs no	8.99	1.25	64.66	0.03
others vs no	0.00	0.00	0.00	0.99
Comorbidities				
diabetes	1.51	0.17	13.55	0.71
dementia	2.10	0.38	11.51	0.39
vegetative state	1.94	0.22	16.81	0.55
CCI	1.65	0.83	3.28	0.15
Cooperation – partial/full vs no	0.42	0.08	2.08	0.29
Aspiration	0.34	0.06	2.11	0.25
Height	1.01	0.97	1.05	0.78
Knee height	1.004	0.92	1.10	0.93
IBW	1.01	0.94	1.07	0.87
MV method – VC-IMV vs others	26.89	0.00	2915387.85	0.58
Pressure support	1.13	0.83	1.54	0.44
PEEP	0.09	0.0001	702.1	0.60
FiO_2_	1.01	.91	1.13	0.85
f	0.98	0.81	1.18	0.81
V_T_	0.00	0.00	1766.48	0.28
V_T_/IBW	1.01	0.70	1.44	0.98
RSBI	1.02	0.97	1.07	0.46
Weaning from O_2_	0.02	0.0001	23.40	0.28
Weaning from catheter	0.23	0.027	2.02	0.18
Weaning from zonda	0.26	0.03	2.26	0.21
Decanulation	0.03	0.0001	57.88	0.36
e-PMV duration – total	0.99	0.98	1.003	0.12
Length of stay in GRC				
Duration of weaning from e-PMV	0.99	0.96	1.01	0.29
Duration from admission to GRC to complete weaning from e-PMV	0.99	0.98	1.005	0.19
Duration since complete weaning from e-PMV to endpoint event (death/ discharge/ rec MV)	0.99	0.98	1.004	0.15
Recurrent MV – after weaning from e-PMV	267.10	0.06	1116667.61	.19

*Abbreviations: CCI – Charlson Comorbidity Index; CI – confidence interval; e-PMV – extremely prolonged mechanical ventilation; FiO_2_ – fraction of inspired oxygen; f – breathing frequency; GRC – geriatric rehabilitation center; IBW – ideal body weight; HR – hazard ratio; PEEP – positive end-expiratory pressure; RSBI – rapid shallow breathing index; VC-IMV – volume control intermittent mandatory ventilation; V_T_ – tidal volume; V_T_ – tidal volume;

**Table 4:** Multivariate Cox’s proportional hazard regression analysis for potential predictors of in-hospital mortality in older adults (N = 73)*

Characteristic	Time-independent (unadjusted), HR (95% CI)	Time-dependent (unadjusted), HR (95% CI)	Time-dependent (adjusted), HR (95% CI)
Age	1.13 (1.008-1.26) *P* < 0.035	1.13 (1.008-1.26) *P* < 0.035	1.36 (0.79-2.35) *P* < 0.26
Charlson Comorbidity Index	1.65 (0.83-3.28) *P* < 0.15	1.65 (0.83-3.28) *P* < 0.15	1.65 (0.52-5.27) *P* < 0.39
Tracheal injuries	*P* < 0.09	*P* < 0.09	*P* < 0.32
Extremely prolonged mechanical ventilation duration	0.99 (0.98-1.003) *P* < 0.12	0.99 (0.98-1.003) *P* < 0.12	1.01 (0.99-1.02) *P* < 0.49
Duration since complete weaning to endpoint	0.99 (0.98-1.004) *P* < 0.15	0.99 (0.98-1.004) *P* < 0.15	0.96 (0.90-1.01) *P* < 13

*Abbreviations: CI – confidence interval; HR – hazard ratio.

### Discharge disposition

Among the 67 participants who survived to discharge from our GRC after successful weaning from e-PMV, 43 participants (58.9%) who remained with tracheostomy were transferred to facilities for complex nursing care, while 6 (8.2%) were transferred to different external hospitals (of them, 2 with recurrent MV after weaning from e-PMV) or nursing facilities. Eighteen participants (24.7%) were successfully decanulated and discharged to their private homes.

## Discussion

Our study is unique in that we looked specifically at the elderly population of patients post e-PMV. In our study, patients weaned from e-PMV had a relatively high survival rate: 67 out of 73 (91.7%) survived to discharge. Other studies that examined survival among elderly patients weaned from PMV demonstrated similar and lower survival rates. Wu et al (13) observed a survival rate of 84.9% among patients post-PMV in Taiwan, while Mamary et al (16) reported the survival of 81%.

Dermot Frengley et al (9) showed that the likelihood of successful weaning after the age of 65 decreased with age. The best predictors for weaning success were lower comorbidity burden and a less severe illness (9), and survival among weaned patients was only 36.5% (9). Yet, Mamary et al (16) found post-discharge survival in weaned patients to be 75% and 59% at 1 and 3 years, respectively.

In our study 26.9% of weaned patients were discharged directly to their homes. Dermot Frengley et al (9) found 23% of their patients to be discharged home.

Other studies found increasing age as a predictor of lower mortality. However, our patients were older, and yet compared with other studies, had lower mortality. The average age of weaned patients in our study was 78 years, whereas in other studies it ranged from 60 to 72.5 years (9,13,18).

We could not find any independent predictive factors that were time dependent. However, univariate analysis indicated age and the presence of tracheomalacia as predictive factors for increased mortality.

There is a paucity of studies examining the short-term outcomes among elderly patients (17,20-22) who were liberated from e-PMV. One such study, by Mamary et al (16), in 182 participants, found in-hospital mortality (19%) to be related to a higher Charlson comorbidity index score and Acute Physiology and Chronic Health Evaluation (APACHE) II score on admission. However, their study population differed significantly from ours. The mean age was 64.1 years, as opposed to 78 years in our study, and the mean hospital length of stay was only 55 days, compared with 145.5 days in our study (16). Aboussouan et al (19) demonstrated similar results.

Out of all participants in our study, about 11.0% needed recurrent MV during their intrahospital admission after successful weaning from e-PMV. To the best of our knowledge, our study provides the first empirical estimation of the recurrent MV rate among older adults with significant comorbidities during their intrahospital admission after successful weaning from e-PMV.

When compared with other reports, our study population was characterized mainly by older age and probably much lower functional status before the start of mechanical ventilation. As a result, our patients were hospitalized and ventilated for much longer periods, a fact that inevitably affected mortality and outcome.

There are a few limitations to this study. This was a retrospective analysis of patients transferred to a single GRC. However, this GRC is a typical and representative one in Israel (17,18). Thus, the potential risk for selection bias is low. In addition, due to incomplete database, some variables, such as the patient’s functioning level before they succumbed to e-PMV (22), or daily protein intake (23,24) were not included in our analysis. Finally, long-term outcomes (eg, all-cause mortality) may be more meaningful and helpful in providing patients with realistic outcome expectations. In conclusion, we did not find an independent predictive factor that could time-dependently predict mortality in elderly patients after e-PMV. These results may suggest that even at the extreme of age, elderly patients with significant comorbidities may have a meaningful chance for successful weaning from e-PMV, and thus should undergo weaning attempts.
